# Applications of Computer Intelligence in Hydrogen Production

**DOI:** 10.1021/acsomega.5c01602

**Published:** 2025-07-29

**Authors:** Hamza Sethi, Iftikhar Ahmad, Maryam Mahsal Khan, Ahmed Qazi, Asad Ayub, Muhammad Zulkefal, Meshal Shutaywi

**Affiliations:** † School of Chemical and Materials Engineering, 66959 National University of Sciences and Technology, Islamabad 44000, Pakistan; ‡ Department of Computer Science, 66937 CECOS University of IT and Emerging Sciences, Peshawar 25000, Pakistan; ¶ Department of Energy and Process Engineering, Norweigian University of Science and Technology (NTNU), 8900, 7491 Trondheim, Norway; § Department of Mathematics, College of Science and Art King Abdul Aziz University, P.O. Box 344, Rabigh 21911, Saudi Arabia

## Abstract

In response to environmental degradation and diminishing fossil fuel reserves, there is an urgent global shift toward sustainable and cleaner energy solutions. Hydrogen has gained importance as an alternative fuel due to its low carbon emissions and high combustion energy, in addition to its capacity for efficient renewable energy storage and transport. This paper presents a comprehensive review of various hydrogen production methods, including water splitting, hydrocarbon reforming, and biological decomposition, and evaluates the integration of machine learning techniques into these processes. By applying intelligent algorithms, the study examines key performance indicators, such as hydrogen yield, gas quality, production cost, and overall efficiency. By leveraging predictive modeling, real-time monitoring, and adaptive control systems, computer intelligence enables the optimization of operational parameters and improvement of energy conversion efficiencies. The findings underscore the pivotal role of machine learning in optimizing production processes, thereby enhancing both the sustainability and the economic viability of hydrogen as a clean energy source.

## Graphical abstract

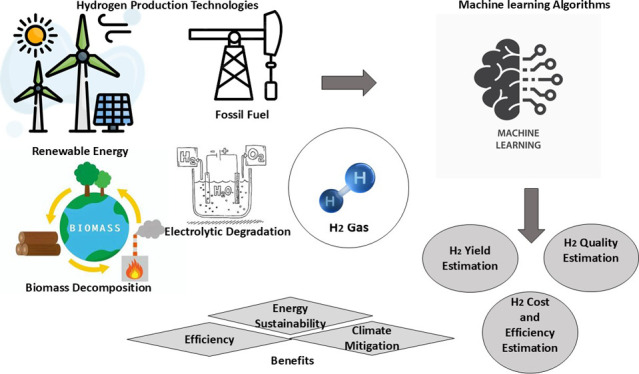

## Introduction

With the degradation of environmental quality due to the unbridled use of fossil fuels and the unsustainable nature of these fuels, the world is shifting toward environmentally friendly renewable energy sources for power generation, namely solar energy, geothermal energy, hydro energy, wind energy, bioenergy, sound energy, and tidal energy.^1^ The main reason for switching from conventional energy to renewable energy is that conventional sources have a detrimental impact on climate and environment.^2^. In addition, energy costs are continually increasing due to fossil fuel depletion; therefore, the need for cheap, clean, and renewable sources of energy has increased over the years.

A few of the renewable energy sources have matured and have been used for power generation for decades, such as solar and wind energy. The major issue with regard to these renewable sources of energy is their unpredictable nature and storage issues.^2^ There are many energy storage mediums, such as batteries, thermal storage, supercapacitors, hydrogen-based systems, etc. The primary obstacles for most energy storage mediums are the risk of uncontrollable energy release, the requirement of a longer charging time, and the inefficiency in maintaining stored energy for longer periods,^2^ which is the focus of many research studies nowadays.

Hydrogen, the most abundant element in nature with a very high calorific value of 120–142 MJ/kg, higher than petrol, diesel, and natural gas, can be used as one of the most efficient and clean energy carriers^3^ and an alternative fuel source. Naturally, hydrogen exists bonded to other elements, requiring it to be produced through a variety of methods, principally classified as thermochemical and biological. Currently, the majority of hydrogen produced worldwide comes from fossil fuels through thermochemical methods, including steam methane reforming (SMR), partial oxidation (POX), and autothermal reforming (ATR), which are well-developed methodologies with high efficiency but are carbon-intensive.^4^ Cleaner alternatives include water splitting through electrolysis (such as alkaline, PEM, and solid-oxide electrolysis) and thermolysis,^5,6^ which uses high-temperature methods mostly to drive thermal chemical cycles to reduce static requirements; but thermolysis is at the moment impractical. The development of renewable hydrogen has gained traction through biomass and water, and among biological methods such as dark fermentation, photofermentation, and biophotolysis^7,8^ have been possible environmentally benign alternatives, which were perhaps the mildest of all methods, though these methods have limited scalability and yield.^9^ The chart in Figure 1 illustrates that hydrogen production is now mainly dependent on natural gas reforming and oil, the share of which is above 75%. This scenario calls for the immediate advent of green alternatives to hydrogen production.

**Figure 1:**
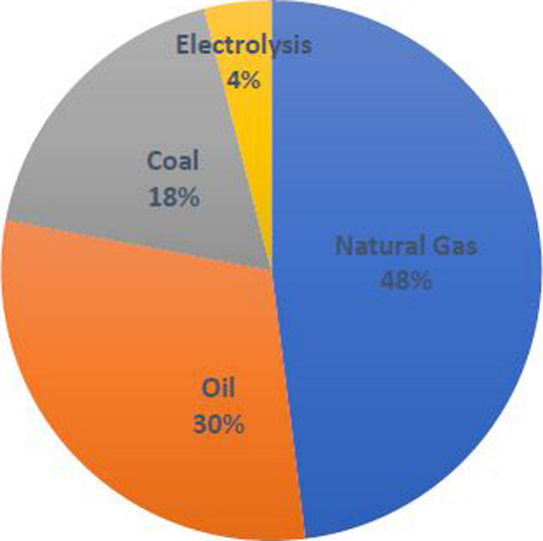
Hydrogen production sources.^10^ Adapted with permission from Ji and Wang. Review and comparison of various hydrogen production methods based on costs and life cycle impact assessment indicators. *Int. J. Hydrogen Energy* **2021**, *46*, 38612–38635. Copyright 2021 Elsevier.

However, in the production of hydrogen, there are several other technical concerns, such as storage, transportation, safety, utilization, and most importantly, production costs, that are associated with the widespread adoption of hydrogen.^11–13^ Many researchers are investigating sustainable and environmentally friendly methods for producing green hydrogen.^14^ Although green hydrogen production methods like water electrolysis, thermochemical cycle, and other methods offer a cleaner alternative, these methods face challenges related to cost, efficiency, and scalability. Kadam et al. investigate three prominent hydrogen production methods: steam reforming, electrolysis, and Cu–Cl cycles, focusing on their efficiency and environmental impacts,^6^ CU-CL cycles is an emerging technology with the lowest environmental footprint, yet the energy efficiency is close to 43%. Moreover, Gabriel et al. also studied these three processes in integration with nuclear and solar energy focused on CAPEX, operational expenditure, and levelized cost of hydrogen (LCOH).^15^

There is a critical research gap in optimizing hydrogen production processes to improve efficiency, reduce operational costs, and minimize environmental impact, especially in renewable-based systems. Traditional optimization methods often fail when dealing with the complexity and nonlinearity of hydrogen production systems.^16^ Artificial intelligence (AI), particularly machine learning (ML), provides a powerful toolkit to bridge this gap.^16^ Machine learning models are developed to discover patterns in data and provide predictions or assessments without the need for explicit programming.^17^ Diverse types of machine learning models are available, such as supervised, unsupervised, and reinforcement learning models.^18^ Depending upon the nature and type of data, ML models are a helpful tool in decision-making as they have been used in pharmaceutical, petroleum, manufacturing, designing, predictive maintenance, optimization, automation, and other domains in industry.^19^ AI enables real-time monitoring, predictive modeling, and adaptive process control, facilitating better decision-making in dynamic environments.^20^

The hydrogen production process, like many other chemical processes, is becoming easier, faster, and even more efficient through the use of new AI techniques,^21^ which are the basis of Industry 4.0, the fourth industrial revolution.^22^ The ML techniques are useful in many ways, such as for investigating the impact of operating parameters on hydrogen production. It is also very important to control a chemical process in order to ensure stability and higher efficiency; ML techniques are very useful to optimize the process in real-time as well as in process automation. These ML techniques are also very useful for predicting the rate of hydrogen production, gas composition, conversion rates, total annual costs, thermal efficiency, production plant’s capacity, and remaining useful lifetime. Utilizing these modern techniques can help improve the overall process in a short amount of time with higher efficiency.

Previous reviews have studied hydrogen production methods and associated computational techniques. For instance, Nikolaidis and Poullikkas provided a general overview of hydrogen production pathways, but did not focus on particular elements of AI integration into the system.^2^ Ardabili et al. studied computational intelligence for hydrogen modeling, but did not have a comparative study on ML methods.^23^ More recently, Mert provided a review of how deep learning was utilized for solar hydrogen systems, but only referenced specific case studies.^21^ Similarly, Shahin et al. investigated artificial intelligence (AI) and LLM tools to analyze challenges in the development of hydrogen energy, including production, storage, distribution, and policy making, which was insufficient to analyze and compare production, yield, quality, and cost estimation using AI.^24^ While the contributions of the very recent studies are valuable, they often fail to include general comparisons of ML methods across hydrogen production pathways, clarify the distinction between thermochemical and biological systems, or the practical considerations for deploying ML models. In this review, we seek to address all of the above, while providing a comprehensive and comparative evaluation of machine learning applications in hydrogen production, especially regarding model performance, methodological variety, and future research possibilities.

This paper provides a comprehensive review of the application of artificial intelligence (AI) techniques, namely machine learning (ML), in hydrogen generation methods, improving performance, and reducing costs. These results indicate that AI has the capacity to revolutionize and put hydrogen at the center of our sustainable energy future. This review is divided into five sections. The first section highlights and explains the types, working methods, and limitations of ML algorithms. The second section explains different hydrogen production methods that exist and their underlying working and drawbacks. The third section explores previous research works with different types of ML algorithms and types of hydrogen production methods used, and the comparison and impact of these studies. Moreover, current challenges and future prospects present the limitations of AI integration into hydrogen production and future directions for application and research. The last section includes the conclusion of this review paper.

## Machine Learning Methods

Machine learning (ML) has the capability to learn from and make predictions based on data. ML’s journey has spanned decades, with pioneering advancements and continuing evolution. Machine learning powers autonomous cars, natural language processing, recommendation systems, and image and audio recognition. Model selection depends on the problem, data type, and desired outcome. Researchers continually innovate to enhance machine learning’s capabilities. A general ML model consists of input data features and a learning algorithm that collectively enable the model to learn from data and make predictions. ML algorithms are categorized into supervised, semi-supervised, unsupervised, and reinforcement learning.^25^

In supervised learning, the data set includes labeled outputs. The algorithm learns from the labeled data and creates a linear/nonlinear mapping of inputs to outputs. Some algorithms in this category include Linear Regression, Gaussian Process Regression, Support Vector Machines, Logistic Regression,^26^ ANN,^21,27,28^ Decision Trees, and Random Forests. The parameter space of these ML algorithms can be trained with different strategies, e.g., ANN-based solutions can be derived by using training algorithms such as the conjugate gradient algorithm, back-propagation algorithm, Bayesian regularization, and Levenberg–Marquardt algorithm^29^ or using evolutionary-based mechanisms such as GA,^30^ evolutionary algorithms (EA), or particle swarm optimization (PSO).^31^ Moreover, ANN is also integrated with fuzzy logic principles in order to map fuzzy input features, e.g., in ANFIS.^32,33^

Unlike supervised learning, unsupervised learning algorithms identify patterns within the data without any explicit output label. This includes (a) clustering algorithms, e.g., K-nearest neighbors (kNN), K-means, hierarchical clustering; (b) dimensionality reduction algorithms, e.g., principal component analysis (PCA), t-distributed stochastic neighbor embedding (t-SNE).^34^ To further improve the learning process’s precision, semi-supervised learning combines a smaller amount of labeled data with a greater amount of unlabeled data. Examples include semi-supervised SVM and graph-based networks.^35^

Semi-supervised learning is a machine learning method that is between supervised and unsupervised learning. To do this, semi-supervised learning constructs a model with a small amount of labeled data and a larger amount of unlabeled data during the training phase. The main goal of this type of machine learning is to increase the learning accuracy when obtaining a labeled data set is expensive and/or time-prohibitive. Additionally, semi-supervised learning is considered a hybrid approach to reduce data efficiency and improve generalization. In the literature of machine learning, four general approaches to semi-supervised learning are self-training, consistency regularization, graph-based methods, and generative models (e.g., VAEs, GANs). Overall, the typical applications of semi-supervised learning are in computer vision and natural language processing.^35^

While training an agent to interact with the environment and learning from getting feedback in the form of rewards or penalties is known as reinforcement learning (RL). In RL, the objective is to identify a series of acts that maximize the overall reward. Among the algorithms in this category are Deep Reinforcement Learning, Q-Learning, and Policy Gradient Methods. Application areas for reinforcement learning include robots, gaming, and optimization.^36^ Other strategies for improving the stability and accuracy of ML algorithms include ensemble-based learning, such as bagging and boosting. Some of the examples under this category include bootstrap aggregated neural networks, gradient boosting regression tree (GBRT), etc.^37^

Figure 2 shows a subset of popular ML algorithms and their categorization effectively applied in various domains of chemical engineering.^25^ Machine learning has played a vital role in changing the landscape of chemical engineering, from its early applications in process control to today’s optimization and predictive modeling.^22^

**Figure 2:**
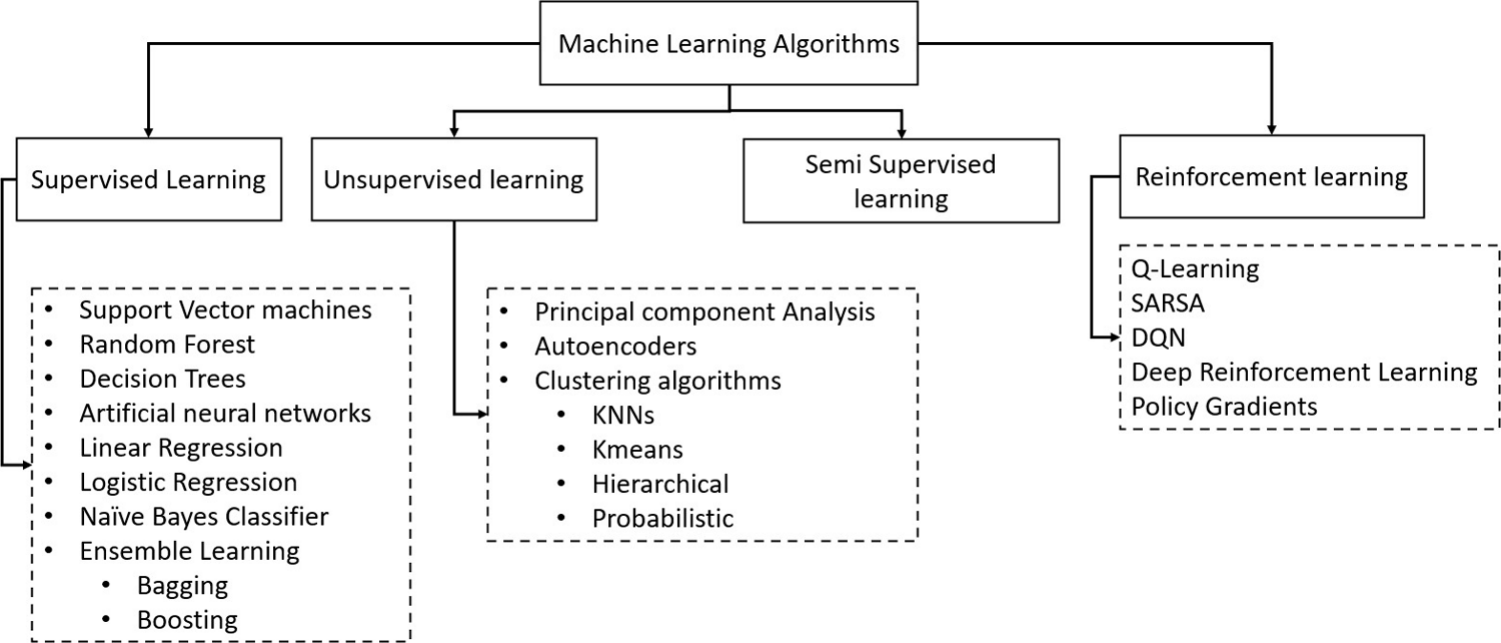
Classification of machine learning algorithms.

Supervised learning models—especially ANNs—are primarily used due to their proven mapping capability of nonlinear relationships. The neural networks, inspired by the human brain’s structure, are highly versatile, excelling in tasks such as speech synthesis, image recognition, and natural language processing due to their ability to learn intricate patterns from input and interconnected layers of neurons.^29^ Although limited in practice, models based on reinforcement and hybrid elements could accomplish dynamic control.^38^ By understanding the types of supervised learning methods, the researcher can confidently select the appropriate algorithm to fit a specific hydrogen production related challenge.

## Methods of Hydrogen Production

Various methods exist for producing hydrogen using both conventional and alternative energy sources. Hydrogen production is divided into two general categories: thermochemical and biological hydrogen production.^10^ Thermochemical methods are high-temperature chemical reactions to extract hydrogen from hydrocarbons or water.^4^ Common methods include steam methane reforming (SMR), partial oxidation (POX), autothermal reforming (ATR), and water electrolysis or thermolysis. Similarly, biological hydrogen production is the production of hydrogen fuel using microorganism cultures in bioreactors via biochemical reactions performed under anaerobic or phototrophic conditions.^39^ The main pathways for this process include dark fermentation, photofermentation, and biophotolysis.^9^ Figure 3 gives a schematic overview of the diverse hydrogen production pathways, highlighting inputs such as feedstock and energy sources, and categorizing the resulting processes into conventional (e.g., SMR, POX) and renewable (e.g., electrolysis, photofermentation). This framework helps us visualize the energy and emission implications of each method.

**Figure 3:**
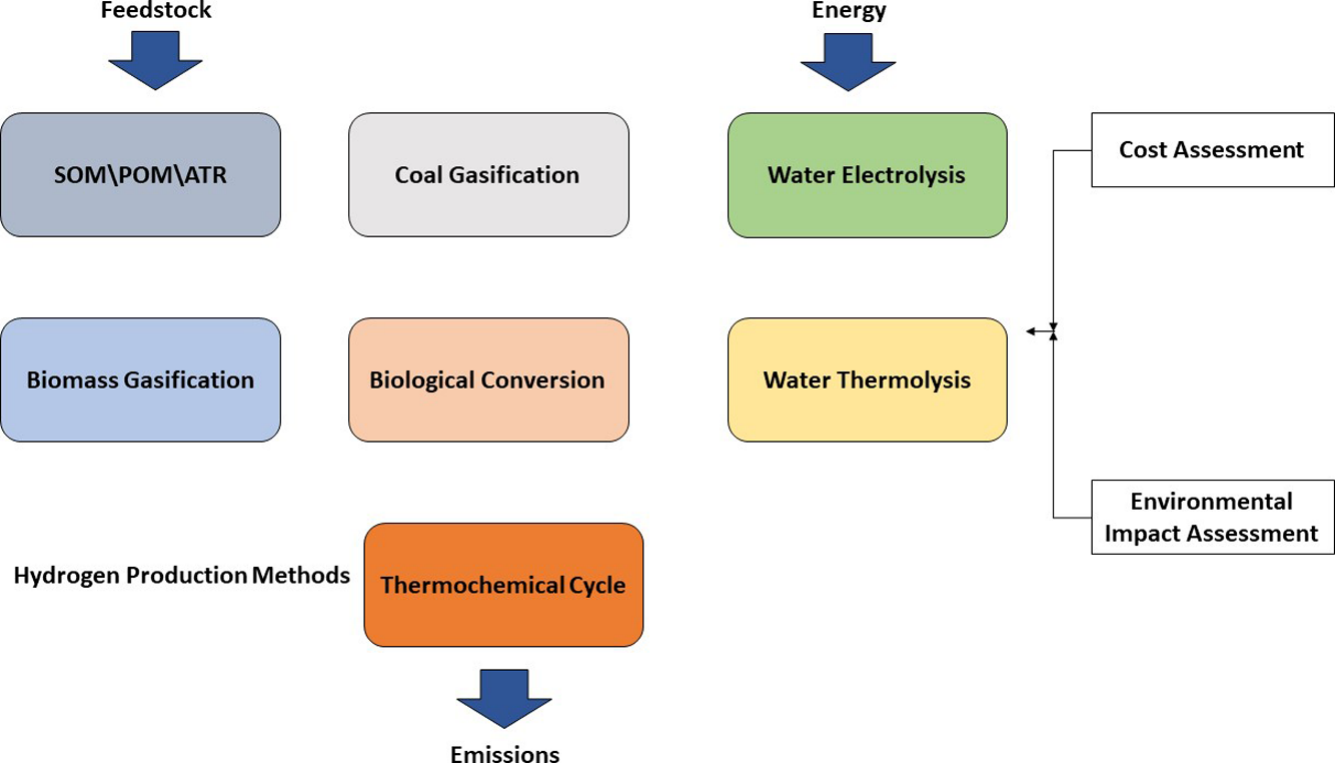
Hydrogen production methodologies.^10^ Reprinted with permission from Ji and Wang. Review and comparison of various hydrogen production methods based on costs and life cycle impact assessment indicators. *Int. J. Hydrogen Energy* **2021**, *46*, 38612–38635. Copyright 2021 Elsevier.

### Water Splitting-Based Hydrogen Production

Water splitting is one of the cleanest methods to produce hydrogen. There are different processes to achieve water splitting, such as electrolysis, thermolysis, and thermochemical cycles.^40,41^ Hydrogen is a clean source, the processes used to produce hydrogen are not clean, so using renewable energy resources for water splitting is one of the cleanest methods.^42^

Water splitting can be done using both electrical and thermal energy. Water splitting done using electrical energy is called electrolysis. The alkaline and electrolysis process can be subdivided into two types: solid oxide electrolysis (SOE) and proton exchange membrane (PEM). Water electrolysis is a very energy-intensive process, and efforts are being made to increase the electrolyzer efficiency as well as the total electrolysis plant efficiency.^2,43^

Water splitting done using thermal energy is known as thermolysis. The thermolysis process requires very high temperatures up to 4000 °C, which is not very practical. Using thermochemical cycles, this high temperature requirement can not only be reduced, but the process can be made much more effective, as different reactions take place in separate reactors and the two products, hydrogen and oxygen, do not need to be separated at the end of the process.^8^

### Hydrogen Production through Hydrocarbon Reforming

One of the predominant methods for producing hydrogen involves the conversion of hydrocarbon fuels using reforming techniques such as partial oxidation, steam reforming, and autothermal reforming.^42^

Steam reforming (SR) is a catalytic conversion of hydrocarbon fuel and steam to hydrogen and carbon oxides. To produce high-purity hydrogen, high temperature, pressure up to 3.5 MPa, and steam-to-carbon ratios (S/C) of 3.5 are usually considered as the operating parameters for steam reforming. The process of steam methane reforming involves desulfurization, if there are any organic sulfur compounds present in the feedstock, reforming, heat recovery, shift conversion, and a pressure swing adsorption system.^2,44,45^

Partial oxidation (POX) is another well-developed method, especially for a heavy hydrocarbon feed. In this method, hydrogen and carbon oxides are produced by the conversion of oxygen, steam, and hydrocarbons. The partial oxidation method can be generally classified into two types: catalytic process and noncatalytic process. The catalytic process operates at 950 °C. Thermal efficiency of the catalytic process ranges from 60% to 75%. POX is a highly cost-intensive process, and the two main reasons are the oxygen plant and desulfurization.^6^

A hybrid method that involves the simultaneous occurrence of SR and POX is known as autothermal reforming. In autothermal reforming, the heat is provided by the exothermic POX, and the increase in hydrogen production is due to endothermic SR.^2,46^

### Hydrogen Production through Biological Methods

Biomass is another source of clean energy that can be used to produce hydrogen without any environmental impact. Plants and animals, such as energy crops, wood, animal waste, municipal waste, etc. are the sources of biomass for the production of hydrogen through thermochemical and biological processes.^42^

The thermochemical process is fast and offers a higher yield of hydrogen. Biomass pyrolysis and gasification are the main methods that can be used for thermochemical hydrogen production. Biomass pyrolysis can be done at temperatures ranging from 650 to 800 K and pressures of 0.1–0.5 MPa. Where the temperature and pressure requirements for biomass gasification are 500–1400°C and 33 bar, respectively, depending on the final application of produced syngas and plant scale.^47,48^

On the other hand, less energy-intensive and highly sustainable biological processes for the production of hydrogen have gained a lot of attention over the last several years. Dark and photofermentation, sequential dark and photofermentation, and direct and indirect biophotolysis are some of the methods used for biological hydrogen production.^2^

Several comparative studies have established a basis for measuring hydrogen production on industrial scales. For example, more recent studies have conducted comparative assessments of SMR, electrolysis, and Cu–Cl cycles, which also assessed energy intensity and environmental impact,^6^ and it showed that multicriteria analysis is essential for choosing ideal methods. Also, the techno-economic review by^15^ emphasized the scalability and cost-wise feasibility of clean hydrogen systems, which aligns with this paper’s focus on cost modeling and optimizing performance driven by ML.

Recent developments have greatly expanded the area of hydrogen research, particularly with the use of AI-based tools. The research conducted in ref 24 utilized the promise of large language models (LLMs) to identify the non-numerical roles for LLMs in hydrogen energy systems for improved data interpretation, process simulation, and system design. It also represents a discernible trend where advanced AI tools are evolving beyond numerical modeling into the area of knowledge synthesis and decision support for hydrogen technologies. Other work has recently investigated the hydrogen generation predictive modeling capabilities. For example, ref 49 utilized a smart connectionist model was used to predict hydrogen output for formic acid dehydrogenation with extremely promising predictive accuracy and robustness under highly complex reaction conditions. A comparative analysis, like the one performed in ref 42 to name just one of many, helps support comparative hydrogen production route identification and evaluation work across the categories of process type, feedstock, and environmental impact, which is imperative to contextualizing and differentiating the context for ML applications for hydrogen production across diversified pathways.

Overall thermochemical processes are inherently more mature and more efficient but they are not clean unless they use renewable energy to power them and are carbon-intensive processes. Biological processes are environmentally friendly and utilize mild temperatures and pressures, but they suffer from a scale and efficiency. Knowing the material differences of these pathways will enable the best decision to be made for production pathway selection when coupled with AI optimization.^42^

## Applications of Machine Learning in Hydrogen Production

ML techniques are widely used to make the processes faster and more efficient. Several research publications are focused on the integration of ML techniques with hydrogen production processes for different purposes, such as predicting the impact of operating parameters on hydrogen production, investigating the hydrogen production rate, estimating the compositions and concentrations, estimating costs and efficiencies, etc. This section of the review provides a thorough survey of 29 research articles in which different ML techniques were utilized for predicting different aspects of the hydrogen production process. This section is divided into three categories as described in Figure 4, where (1) H_2_ yield estimation, (2) H_2_ quality estimation, and (3) H_2_ cost and efficiency estimation are being done.

**Figure 4:**
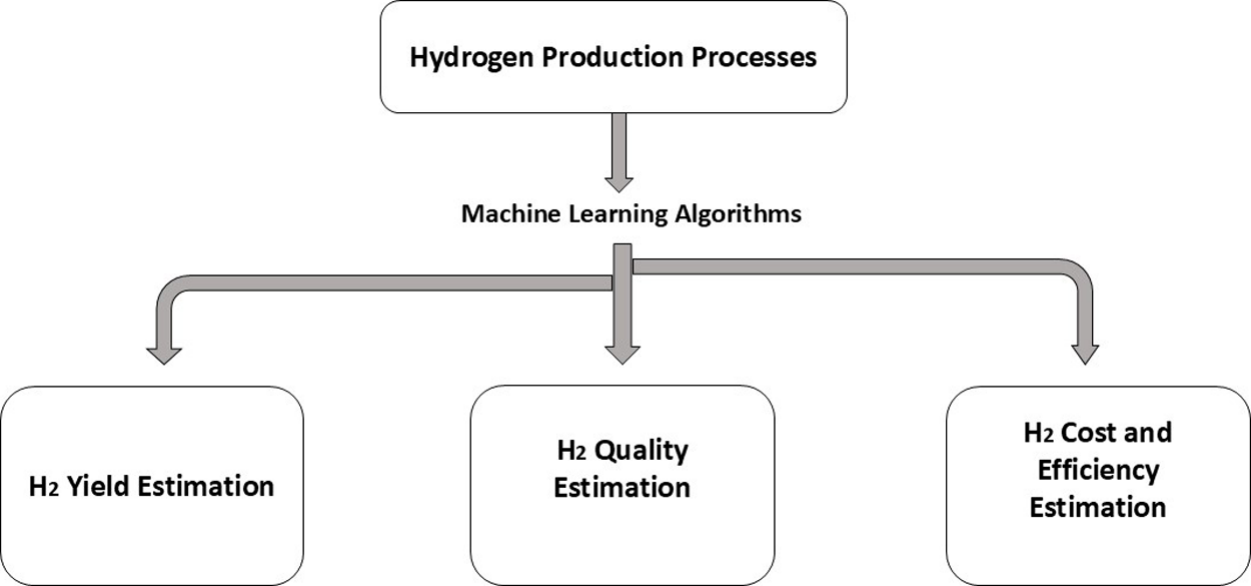
Application of ML techniques to hydrogen production processes.

### Hydrogen Yield Estimation through Machine Learning

This subsection includes key findings of different research articles focused on the estimation of hydrogen yield and hydrogen production rates utilizing ML techniques.

Mullai et al.^50^ studied the impact of operating parameters on hydrogen production in a laboratory-scale Anaerobic Sludge Blanket filter (ASBF). An ANN-based model was developed for prediction, where input variables to the ANN model were pH, acidity, glucose concentration, biogas production rate, and hydrogen production rate was the output of the model. The value of the coefficient of determination *R* ^2^ was 0.9981.

Alsaffar et al.^51^ developed an ANN to predict the production rate of hydrogen from methane through thermo-catalytic decomposition. The impact of the input variables (reaction temperature, catalyst weight, calcination temperature, c, time of stream, calcination time, and specific volume) on hydrogen production was investigated through sensitivity analysis. The Levenberg–Marquardt trained ANN with a model topology of 7-16-1, generating *R* ^2^ and MSE values of 0.953 and 0.03, respectively, was found to be in much stronger agreement with the measured values as compared to the Bayesian regularization trained network.

Hydrogen production through the steam reforming process using bio-oil and glycerol is gaining wide acceptability as these can be produced in huge amounts as a byproduct of biodiesel production and the pyrolysis process. Mageed et al.^52^ predicted hydrogen production from steam reforming of bio-oil and glycerol. In their study, they used an ANN model using the backpropagation training algorithm for predicting hydrogen production. The effect of various process parameters, such as weight hourly space velocity, reaction temperature, steam-to-carbon molar ratio, catalyst weight, feed flow rate, catalyst loading rate, and glycerol–water molar ratio, on hydrogen production was also investigated using sensitivity analysis. The ANN was trained using the Levenberg–Marquardt (LM) algorithm and Bayesian regularization (BR). The optimized LM and BR trained ANN generated an *R* ^2^ value of 0.998 and MSE values of 3.024 × 10^–24^ and 6.22 × 10^–15^, respectively.

As biological hydrogen production is one of the most efficient hydrogen production methods. Therefore, cheap and practical methods for hydrogen determination are much needed. Rosales-Colunga et al.^53^ developed an ANN using a conjugate gradient training algorithm for the estimation of hydrogen production in a fermentative process. The estimation was performed based on the online measurements of dissolved carbon dioxide, oxidation–reduction potential, and pH in genetically modified *E. coli* fermentation. The value of the coefficient of determination *R* ^2^ was 0.955.

Moreno Cardenas et al.^54^ produced hydrogen from the dark fermentation of coffee mucilage in a co-digestion system. In this study, they used an ANN and a fuzzy logic model approach to predict the hydrogen yield of this system. Both systems performed well in the prediction of the hydrogen yield. The input factors used in this study were acidification time, substrate ratios, pH, chemical oxygen demand, and temperature. The fuzzy logic model showed better prediction results with an *R* ^2^ value of 0.9508, where the *R* ^2^ value of the ANN model was 0.8369.

Mert^21^ estimated the hydrogen production rate of a PEM electrolyzer using solar radiation data. The estimations were achieved using the ANN, ANFIS, MLR, EAP, and DL models. The day length, sunshine duration, soil temperature, extra terrestrial solar radiation, solar radiation, and relative sunshine fraction were used as input variables, and the amount of production of hydrogen was the output of the prediction models. Agnostic **deep learning** model structure 5-100-10-1 outperformed all the other models with the coefficient of determination *R* ^2^ value of 0.9626, followed by the EAP model with the *R* ^2^ value of 0.9476, while the ANFIS model had the smallest value of *R* ^2^ among all the models.

El-Shafie^55^ used an ANN model to predict hydrogen yield using anaerobic fermentation and compared the results with the traditional backward design (BWD) approach. The ANN outperformed the BWD approach with a maximum error of 10%. The input variables for the ANN model were initial substrate, initial medium pH, and reaction time. The best model topology for the ANN was 3-6-4-2-1, and the *R* ^2^ value was 0.9847.

Zhao et al.^56^ developed and compared four machine learning models: RF, ANN, GPR, and SVM. These models were developed to predict the hydrogen yield of the supercritical water gasification process of biomass. The input parameters for the model were carbon, hydrogen, oxygen, ash content, biomass concentration, gasification temperature, reactor pressure, and residence time. The Random Forest model outperformed all the remaining three models with MAPE, RMSE, and *R* ^2^ values of 4.1779% 0.1065, and 0.9782, respectively.

Hossain et al.^57^ used SVM and GPR to model the production of hydrogen from organic waste effluents, i.e., activated sludge waste and palm oil mill effluents. The SVM and GPR models were incorporated with different kernel functions. Hyperparameter grid searches for both models were performed across different features and cross-validations. The input parameters for the models built for hydrogen production from palm oil mill effluents were reaction temperature, influent COD, and pH, while the input parameters for hydrogen production from activated sludge waste models were temperature, pH, and glucose concentration. It was concluded on the basis of the values of *R* ^2^ that the SVM models were not able to model the hydrogen production efficiently, and the GPR models had the best performance for both types of effluents, with the *R* ^2^ values of 0.999.

As hydrogen production depends on a number of factors, it is difficult to maintain efficient production all the time. Hu et al.^58^ proposed a forecasting model based on Discrete Gray Method (DGM) and Gradient Boosting Regression Tree (GBRT), GBRT-DGM­(1,n), to forecast the hydrogen production yield of a biomass-based process. The results of this forecasting model were also compared with SVM, GBRT, DGM­(1,n), ARIMA, and particle swarm optimization–backpropagation neural network (PSO-BPNN), but the proposed model outperformed all the other models. The values of C, O_2_, H_2_, ash, bioconcentration, gasification temperature, and residence time were used as input variables. The *R* ^2^ value was around 0.90, and the value of MAPE was between 10% and 15%.

Khan et al.^59^ developed four machine learning models, ANN, ELT, SVM, and GPR, for the prediction of hydrogen production based on supercritical water gasification of sewage sludge. The Particle Swarm Optimization technique was used to optimize the production process. The values of temperature, pressure, time, fixed carbon, ash, moisture content, H_2_, O_2_, N_2_, C, and S were used as input parameters. The ELT-PSO model outperformed all the other models with an *R* ^2^ value of 0.997 and an RMSE value of 0.093. The ELT-PSO model was also used to develop a graphical user interface (GUI) to easily calculate the hydrogen yield.

Li et al.^28^ investigated the production of hydrogen through the biomass gasification process. In their work, they developed an ANN model to predict the behavior of biomass for hydrogen production and the production of syngas, which was based on experimental data. The input variables for the ANN model were steam/biomass (S/B) ratio, ash content, moisture content, biomass particle size, reaction temperature, equivalence ratio, amount of C, H, and O, and the output variables were gas composition and hydrogen yield. The model was validated using the experimental data from the literature review. The RMSE error value, when the S/B ratio is 3.0, was 2.09.

Ghasemian et al.^39^ investigated an anaerobic migrating blanket reactor (AMBR) for the production of hydrogen and water treatment. A backpropagation ANN was developed to predict the performance of the AMBR for hydrogen production and COD concentration. In this study, it was found that the Organic Loading Rate (ANN input variable) had a direct impact on the hydrogen production rate. A sigmoid transfer function was used to map the input layer to the hidden layer, and a pure linear transfer function was used to map the hidden layer to the output layer. The value of *R* ^2^ for the ANN model was 0.92.

Yogeswari et al.^60^ used an Anaerobic Sludge Blanket Filter (ASBF) reactor to produce hydrogen from confectionery wastewater. ANN was used to predict the effluent COD concentration and hydrogen rate, which were validated with the experimental data. The best model topology was 4-12-4-2. The input parameters used for the ANN were time, influent COD, effluent pH, and volatile fatty acids. The *R* ^2^ value of the model was 0.996 and the average percentage error (APE) was 0.0004.

Mu and Yu^79^ developed a neural network GA (GA-NN) model to predict the performance of a granule-based hydrogen production through a UASB reactor. The input parameters for the model were hydraulic retention time (HRT), organic loading rate, and influent bicarbonate alkalinity. The model output parameters were HPR, hydrogen concentration, hydrogen yield, propionate, effluent aqueous products including acetate, effluent total organic carbon, butyrate, valerate, and caproate. The value of *R* ^2^ of the model for hydrogen yield was 0.843, which showed that it is possible to use the GA-NN model for the prediction of the performance of a UASB reactor.

Ghasemzadeh et al.^61^ developed an ANN model, trained with experimental data and optimized using the Levenberg–Marquardt algorithm for methanol steam reforming. Their work represented a black-box method for methanol steam reforming in a silica membrane reactor and a traditional reactor. The ANN model was used to predict the behavior of the operating parameters, which include: pressure difference, reaction temperature, steam-to-methanol ratio, and gas hourly space velocity. The output variables of the model were total hydrogen yield, Hydrogen composition, Methanol conversion, carbon monoxide composition, hydrogen recovery, and carbon monoxide recovery. The configuration of the best ANN model was 4-15-6, and the value of the coefficient of determination *R* ^2^ was 0.9986.

Azaman et al.^62^ investigated hydrogen production from glycerol steam reforming using nickel-loaded zeolite. ANN, trained using the Levenberg–Marquardt algorithm, and Response Surface Methodology (RSM) were used for hydrogen prediction. In this study, the prediction models were compared, where ANN performed much better than the RSM, with the *R* ^2^ and MSE values for hydrogen production being 0.94215 and 4.37916, respectively. The input variables of the prediction model were catalyst weight and glycerol flow rate.

Mohammadidoust and Omidvar^63^ used a Gibbs reactor to produce hydrogen through the hydrothermal gasification method from methane and carbon monoxide. The experimentation, as well as simulations using Aspen Plus software and ANN, was carried out in this study. Both the Aspen Plus and ANN models were developed separately, and the results were compared at the end. Reaction temperature, flow rate of water, and flow rate of biomass were used as input variables to the ANN model. The *R* ^2^ value of the ANN model was 0.9997.

Kargbo et al.^64^ developed a robust bootstrap aggregated neural networks (BANN) model, validated with experimental data, for two-stage gasification of waste wood. The BANN was developed to optimize the operating conditions of the system for high hydrogen yield, high carbon conversion, and low CO_2_. The Levenberg–Marquardt training algorithm was used to train the network, and the input parameters for the network were devolatilization temperature, steam-to-biomass ratio, gasification temperature, carrier gas flow rate, feedstock hydrogen, feedstock carbon, and feedstock oxygen. The BANN performed better than a single hidden layer ANN model. The predicted results of BANN were in good agreement with the experimental data, and the value of the absolute relative error was between 0.01 and 0.05. The *R* ^2^ value of the proposed model was 0.999.

Ayodele et al.^65^ predicted the impact of process parameters on hydrogen production via steam reforming. In his study, he used an ANN model and a nonlinear RSM. The training methods used were scaled conjugate gradient and gradient descent. The activation functions were the hyperbolic training function and the sigmoid function. The input variables were steam partial pressure, methane partial pressure, and reaction temperature. The output variables were methane conversion and hydrogen yield. The sigmoid function with scaled conjugate trained ANNs predicted 89.55% maximal hydrogen yield, and the values of *R* ^2^, MAPE, and MSE were 0.997, 0.199, and 0.121, respectively. The hyperbolic tangent activation function with gradient descent trained ANN model, with the *R* ^2^, MSE, and MAPE values of 0.992, 0.125, and 0.174, respectively, predicted 89.73% of maximal hydrogen, which was slightly greater than the experimental result of 89.51%. The MLP-ANN outperformed the RSM.

Jha et al.^66^ performed fermentation experiments using an Upflow Anaerobic Sludge Blanket (UASB) bioreactor for hydrogen production and removal of chemical oxygen demand (COD). The operating parameters were immobilized cell volumes, hydraulic retention times, and temperatures. In this study, ANN and Response Surface Methodology (RSM) were also used to predict hydrogen yield and COD removal. The results of both techniques were validated with the experimental data; ANN performed the predictions with a much smaller error than the RSM. The value of *R* ^2^ for ANN was 0.99 for hydrogen yield and COD removal. Table 1 presents the overall summary for hydrogen yield estimation while highlighting the hydrogen production methodology, machine learning models used, modeling structures, training algorithms, input and output parameters, performance results, and other related details for each publication.

**Table 1:** Hydrogen Yield Estimation Summary

hydrogen production method	ML type	modeling structure	training algorithm	input variables	output variables	best model performance	remarks	year of publication	references
continuous anaerobic sludge blanket filter	ANN	4-12-4-1		glucose concentration, pH, acidity, biogas production rate	hydrogen production rate	*R*^2^ = 0.9981	the predicted hydrogen production rate was in close agreement with the experimental results	2013	Mullai et al.^50^
thermocatalytic methane decomposition	ANN	7-16-1	Levenberg–Marquardt, Bayesian regularization	catalyst weight, reaction temperature, time of stream, calcination temperature, calcination time, specific volume	hydrogen yield	*R*^2^ = 0.953	Levenberg–Marquardt algorithm performed better than the Bayesian regularization algorithm	2021	Alsaffar et al.^51^
steam reforming of bio-oil	ANN	3-5-1	Levenberg–Marquardt, Bayesian regularization	steam-to-carbon molar ratio, reaction temperature, **space velocity**	hydrogen yield	*R*^2^ = 0.998	Bayesian regularization algorithm performed better than the Levenberg–Marquardt algorithm	2020	Mageed et al.^52^
steam reforming of glycerol	ANN	5-8-1	Levenberg–Marquardt, Bayesian regularization	feed flow rate, reaction temperature, catalyst weight, catalyst loading, glycerol–water molar ratio	hydrogen yield	*R*^2^ = 0.998	Bayesian regularization algorithm performed better than Levenberg–Marquardt algorithm	2020	Mageed et al.^52^
hydrogen production in genetically modified *E. coli* fermentation	ANN	3-12-1	conjugate gradient algorithm	dissolved CO_2_, oxidation reduction potential, pH	hydrogen production rate	*R*^2^ = 0.955	the BPNN successfully predicted the hydrogen yield using only online parameters	2010	Rosales-Colunga et al.^53^
hydrogen production from dark fermentation of coffee mucilage waste	ANN fuzzy Logic model	5-10-1	backpropagation algorithm (BPA)	substrate ratios, chemical oxygen demand, acidification time, pH, and temperature	hydrogen production rate	*R*^2^ > 0.786 (ANN) > 0.848 (FLM)	fuzzy model performed better than the ANN	2020	Moreno Cardenas et al.^54^
hydrogen production from solar-powered system	BPNN, ANFIS, DL, MLR, EAP	5-100-10-1 (DL)	Levenberg–Marquardt	sunshine duration, day length, solar radiation, soil temperature, relative sunshine fraction, extraterrestrial solar radiation	hydrogen production	*R*^2^ = 0.962 (DL)	the most successful model was DL model	2021	Mert et al.^21^
hydrogen production using anaerobic fermentation	ANN	3-6-4-2-1	gradient descent	initial medium pH, initial substrate, reaction time	hydrogen yield	*R*^2^ = 0.984	the ANN model achieved consistent level of accuracy for hydrogen yield within maximum error of 10% and overcomes the limitation of the BWD approach	2014	El-Shafie et al.^55^
hydrogen production via supercritical water gasification of biomass	RF/ANN/SVM/GPR		Bayesian optimization method	oxygen, carbon, hydrogen, gasification temperature, ash content, biomass concentration, reactor pressure, residence time	H_2_ yield	*R*^2^ = 0.978 (RF)	this work provided a precise and interpretable approach for simulation of hydrogen production via SCWG of biomass	2021	Zhao et al.^56^
hydrogen production from palm oil mill effluent	RGPR/SEGPR/DEGPR		different features, k-fold validations, and hyperparameter settings were used to train the models	influent COD, reaction temperature, pH	hydrogen production	*R*^2^ = 0.999	SVM-based models did not show impressive performance in modeling the hydrogen production	2022	Hossain et al.^57^
hydrogen production from waste-activated sludge	FGSVM/RGPR/SEGPR		different features, k-fold validations, and hyperparameter settings were used to train the models	pH, temperature, glucose concentration	hydrogen production	*R*^2^ = 0.980 (FGSVM), 0.999 (RGPR, SEGPR)	SVM-based models did not show impressive performance in modeling the hydrogen production	2022	Hossain et al.^57^
biomass-based hydrogen production	GBRT-DGM (1,n)			C, O_2_, H_2_, ash, bioconcentration, residence time, gasification temperature	hydrogen production yield	MAPE = 10%–15%, *R* ^2^ > 90%	based on the comparison with other forecasting models the GBRT, DGM (1, n) model has the highest precision with least mean absolute error of 0.2	2022	Hu et al.^58^
hydrogen yield from supercritical gasification of sewage sludge	ANN/ELT/SVM/GPR			temperature, fixed carbon, pressure, ash, time, moisture content, H_2_, O_2_, N_2_, C, S	hydrogen yield	*R*^2^ = 0.997 (ELT)	four different types of machine learning techniques with optimum hyperparameters were developed. ELT-PSO outperformed GPR-PSO, SVM-PSO, and ANN	2023	Khan et al.^59^
biomass gasification	ANN	9-2-1		steam/biomass ratio, ash content, moisture content, reaction temperature, biomass equivalence ratio, particle size, amount of C, H, O	gas composition, hydrogen yield	-	the predicted data were in good agreement with the experimental data	2019	Li et al.^28^
biohydrogen production from synthetic wastewater AMBR	ANN	3-9-1	-	organic loading rate	COD concentration, hydrogen production	*R*^2^ = 0.92	the ANN model was well fitted to the experimental data obtained	2019	Ghasemian et al.^39^
hydrogen production from confectionery wastewater	ANN	4-12-4-2	backpropagation algorithm (BPA)	time, influent COD, effluent pH, volatile fatty acids	hydrogen production rate and Effluent COD concentration	*R*^2^ = 0.996	the result of the test data for hydrogen production rate was extremely successful.	2019	Yogeswari et al.^60^
hydrogen production in a UASB reactor	BPFF-GA	3-4-2-10		hydraulic retention time, organic loading rate, influent bicarbonate alkalinity	HPR, hydrogen concentration, hydrogen yield, effluent total organic carbon, effluent aqueous products including acetate, propionate, butyrate, valerate, caproate	*R*^2^ = 0.843	it is feasible to apply a GA–NN model to simulate the performance of the granule-based hydrogen-producing reactor under various input conditions	2007	Mu et al.^79^
hydrogen production in silica membrane reactor	ANN	4-15-6	Levenberg–Marquardt	pressure difference, reaction temperature, steam/MeOH ratio, gas hourly space velocity	total hydrogen yield, CO composition, hydrogen composition, methanol conversion, CO, hydrogen recovery selectivity	*R*^2^ = 0.998	this work represented a black-box theoretical method of MSR reaction in silica membrane reactor compared to traditional reactor via ANN modeling	2018	Ghasemzadeh et al.^61^
hydrogen production using nickel-loaded zeolite	ANN	2-3-2	Levenberg–Marquardt	catalyst weight, glycerol flow rate	hydrogen yield, glycerol conversion	*R*^2^ = 0.9421	ANN performed better than RSM	2015	Azaman et al.^62^
hydrogen production from wheat straw biomass	ANN	3-10-3	Levenberg–Marquardt	reaction temperature, flow rate of water, flow rate of biomass	hydrogen, methane, carbon monoxide	*R*^2^ = 0.999	both the ANN and Aspen Plus performed good enough to predict the experimental data	2022	Mohammadidoust et al.^63^
two-stage gasification for hydrogen production	BANN	7-11-7	Levenberg–Marquardt	steam to biomass ratio, devolatilization temperature, gasification temperature, feedstock hydrogen, carrier gas flow rate, feedstock carbon, feedstock oxygen	gas yield, residue, tar yield, H_2_ yield, CO yield, CO_2_ yield, CH_4_ yield	*R*^2^ = 0.999	the developed BANN model successfully predicted the gas yield and composition	2023	Kargbo et al.^64^
hydrogen production by catalytic steam methane reforming	ANN/RSM	3-17-15-2	scaled conjugate, gradient descent	methane partial pressure, steam partial pressure, and reaction temperature	hydrogen yield, CH_4_ conversion	*R*^2^ = 0.997	ANN model configurations were significantly influenced by the type of training algorithm used, the number of hidden layers, the number of neurons in hidden layers, and the type of activation function. ANN model outperformed the RSM	2021	Ayodele et al.^65^
hydrogen production in an Upflow anaerobic sludge blanket (UASB) bioreactor	ANN	3-4-4-2	backpropagation algorithm (BPA)	hydraulic retention times, immobilized cell volumes, temperatures	biohydrogen yield, COD removal efficiency	*R*^2^ = 0.99	ANN was more reliable than RSM	2017	Jha et al.^66^

The literature clearly shows that ML models, especially ANN and GPR, can predict hydrogen yield with very high accuracy *R* ^2^ > 0.95 across various production methods. Hybrid models offer added precision but require careful tuning. These tools enable early-stage process optimization without the need for extensive experimentation.

### Cost and Efficiency Estimation through Machine Learning

This subsection includes research articles related to hydrogen production costs and efficiency estimation through machine learning.

Steam reforming is currently being used for almost 80% of hydrogen production. Wang et al.^67^ used ANN models for steam methane reforming to predict the hydrogen production rate, total annualized cost (TAC) and molten salt heat duty and a hybrid optimization algorithm was used to solve the optimization problem. The values of steam-to-methane ratio, flow rate of natural gas, reactor temperature, reformer temperature, number of tubes, and tube length of the prereformer were considered as the input variables. The *R* ^2^ value of all the ANN models was 0.9999.

Senthilraja et al.^68^ developed a photovoltaic-thermal (PVT) solar collector-based hydrogen production system and studied the impact of solar collector design, PV module tilt angle, and heat transfer fluid on the performance of the PVT solar collector and hydrogen production rate. The ANFIS technique was used to estimate the electrical efficiency, thermal efficiency, and hydrogen production rate. Cell temperature, time, and solar radiation were selected as input variables. The coefficient of determination *R* ^2^ value of the model was 0.999.

Mehrenjani et al.^18^ designed a novel integrated system with cooling, power, and hydrogen production capabilities in which geothermal energy was used as a heat source, and an LNG stream was used as a heat sink. ANN was used to predict the production rate of hydrogen, exergy efficiency, and total cost. A multi-objective optimization was applied using GA. The linear programming and mapping (LINMAP) method was used for optimal solutions. The values of geothermal fluid mass flow rate, temperature, and pressure were selected as input variables, and the *R* ^2^ value was 0.9998.

Yilmaz et al.^38^ used an ANN on a field programmable gate array to perform the thermo-economic analysis of hydrogen production based on a geothermal water splitting system. The Levenberg–Marquardt backpropagation algorithm was used to train the ANN. The inputs for the model were ambient temperature, geothermal water flow rate, and geothermal water temperature. The output variables were the cost rate associated with exergy for hydrogen production, the cost rate associated with exergy for electricity production, and the payback period. The MSE value of the proposed model was 3.75 × 10^–8^.

Nuclear energy is one of the most significant sources for hydrogen production because of no greenhouse gas (GHG) emissions during the operation and large-scale production capability. The study of the impact of these energy sources on the environment is also of vital importance. Ozbilen et al.^69^ studied the environmental impact of a nuclear-based hydrogen production through thermochemical water splitting using (three-, four-, and five-step) Cu–Cl cycles. A life cycle assessment (LCA) was used to evaluate and reduce the environmental impact of the hydrogen production system. ANN was used to predict the acidification potential, hydrogen plant efficiency, and global warming potential. The input parameters used were the capacity of the plant, plant lifetime, heat input, and electrical input. The *R* ^2^ value of the model was 0.9991.

Aghabashlo et al.^70^ performed the optimization of a photobioreactor for the production of hydrogen via syngas. An ANFIS model was used to develop the objective functions, where input parameters for the model were culture agitation speed and syngas flow rate. Nondominated sorting GA II (NSGA-II) was integrated with the ANFIS model for optimization. The values of MSE, *R* ^2^, and MAPE were 0.1965, 0.9050, and 0.0837, respectively. The method was recommended for the optimization of large-scale photobiological hydrogen production systems.

Machine learning models that are combined with optimization algorithms like Genetic Algorithms (GA) and Nondominated Sorting Genetic Algorithms II (NSGA-II) can make significant contributions to economic modeling and process design. In practical applications and techno-economic assessments, they have very high accuracies (often *R* ^2^ > 0.99), which supports their application to renewable-based hydrogen systems. The overall description of the cost estimation review paper is given in Table 2, which highlights the hydrogen production methodology, machine learning models used, modeling structures, training algorithms, input and output parameters, performance results, and other related details for each publication.

**Table 2:** Cost and Efficiency Estimation Summary

hydrogen production method	ML type	modeling structure	training algorithm	input variables	output variables	best model performance	remarks	year of publication	references
hydrogen production from solar-steam methane reforming (SSMR-MS)	ANN-GA	13-X-1	Lavenberg–Marquardt	flow rate, ratio of steam and methane, reformer temperature, temperature of HWGS reactor, LWGS tube length and the number of tubes	hydrogen production rate, total annualized cost (TAC), and molten salt heat duty	*R*^2^ = 0.999	the computational results have demonstrated the effectiveness and accuracy of the surrogate models in prediction of total annualized cost (TAC), hydrogen production rate, molten salt heat duty, and solar-related cost	2022	Wang et al.^67^
hydrogen production through electrolysis using photovoltaic-thermal (PVT) solar collector	ANFIS	-	Sugeno fuzzy inference system	cell temperature, time, solar radiation	thermal efficiency, electrical efficiency, hydrogen production rate	*R*^2^ = 0.999	the predicted values are in good agreement with the experimental values	2020	Senthilraja et al.^68^
hydrogen production based on geothermal energy	ANN-GA	5-20-1	-	geothermal fluid mass flow rate, T1, Δ*T*pp,ORC2, P12, P14	hydrogen production rate, total cost rate, exergy efficiency	*R*^2^ = 0.999	increasing the geothermal fluid mass flow rate from 60 to 88 improved the hydrogen production capacity by 46.34%	2022	Mehrenjani et al.^18^
geothermal energy -assisted hydrogen production system	ANN	3-8-3	Levenberg–Marquardt backpropagation algorithm	geothermal water temperature, ambient temperature, geothermal water flow rate	cost rate associated with exergy for hydrogen production, Cost rate associated with exergy for electrical production, payback period	MSE = 3.75 × 10^–8^	The results of the ANN model were very close to the actual results	2019	Yilmaz et al.^38^
nuclear-based hydrogen production from copper-chlorine cycle	ANN	4-10-3	Levenberg–Marquardt	plant lifetime, plant capacity, heat, and electrical work input	acidification Potential, global warming potential, hydrogen plant efficiency	*R*^2^ = 0.999	ANN model results agree well with the GaBi 4 software results	2013	Ozbilen et al.^69^
photobiological hydrogen production through multi-objective exergetic optimization	ANFIS	2-X-3	-	culture agitation speed, syngas flow rate	exergy destruction, exergy efficiency	*R*^2^ > 0.9	optimized operating conditions of the bioreactor	2016	Aghbashlo et al.^70^

### Quality Estimation through Machine Learning

Zamaniyan et al.^71^ used an ANN model to predict the process of a hydrogen production plant. The ANN model was trained using the gradient descent algorithm. The model inputs were steam-to-carbon ratio, temperature, pressure, and ratio of carbon dioxide to methane in the feed, and the model outputs were hydrogen mole fraction and CO mole fraction. The coefficient of determination *R* ^2^ value of the model was 0.983.

Ozbas et al.^26^ performed the experiments of biomass gasification of olive pit waste in a steel fixed-bed updraft gasifier with a cyclone separator. Various regression models were developed using LR, SVM, KNN regression, and DTR models trained with the experimental data to predict the hydrogen concentration. The input parameters for these models were time, CO, temperature, CO_2_, O_2_, CH_4_, and heating value. The coefficient of determination *R* ^2^ values for all the models were above 0.90, but the Linear Regression model outperformed all the other models with the *R* ^2^, MSE, and MAE values of 0.999, 0.008, and 0.007, respectively. The results of this study showed that the predictions made by these models for the hydrogen concentration were significant.

Models concentrating on quality usually take advantage of simpler algorithms when the relationship between the inputs and gas composition is relatively straight. To summarize, Table 3 gives a summary of the application in hydrogen production methods, the modeling structure, and all variables associated used in the study. However, the ANN offers better flexibility when there is variability in any dynamic or industrial environment.

**Table 3:** Quality Estimation Summary

hydrogen production method	ML type	modeling structure	training algorithm	input variables	output variables	best model performance	remarks	year of publication	references
industrial hydrogen plant	ANN	4-3-2	gradient descent algorithm	temperature, pressure, steam-to-carbon ratio, CO_2_-to-CH_4_ ratio of the feed	hydrogen mole fraction, CO mole fraction	*R*^2^ = 0.983	ANN successfully modeled the process of hydrogen production plant	2013	Zamaniyan^71^
biomass gasification	LR/KNN/SVMR/DTR			time, temperature, CO, CO_2_, CH_4_, O_2_, and heating value	hydrogen concentration	*R*^2^ = 0.999	LR outperformed all the other models and was significant for predicting H_2_ concentration	2019	Ozbas^26^

### Comparative Analysis of ML Applications in Hydrogen Production

A comparison of the studied research papers in this review reflects patterns in the use of machine learning (ML) approaches across different processes of hydrogen production. Of all the algorithms used, the most frequent is Artificial Neural Networks (ANN), which is preferred based on the type of different nonlinear and complex dynamics of processes. In yield prediction, ANN models achieved high performances with *R* ^2^ ratings reaching often over the ratio of 0.95, particularly when trained using either Levenberg–Marquardt or Bayesian Regularization algorithms. On the other hand, among all cases, GPR was the most accurate in producing models of biological and waste-sourced hydrogen production systems when compared with SVM, reaching up to 0.999 as an *R* ^2^ value. Hybrid models, such as ANFIS combined with particle swarm optimization (PSO) or gradient boosted regression trees (GBRT), improved predictive acumen and robustness at the cost of increased computational effort and careful parameter tuning.

These applications include the hydrogen yield, total annual cost (TAC), thermal efficiency, and exergy efficiency. The model-improved efficiency levels using ANN and ANFIS for cost and efficiency estimation using optimization techniques such as GA and NSGA-II. They were found to be highly efficient at predicting such techno-economic parameters as hydrogen yield, TAC, thermal efficiency, and exergy efficiency, with that regarded to experimental or simulated data. For example, for gas quality prediction, simpler regression models such as Linear Regression (LR) and Decision Tree Regression (DTR) were much better able to explain their linear or mildly nonlinear input–output relationships, such as that between gas composition or temperature and hydrogen mole fraction as the output quality indicator.

From a broader perspective, the selection of ML algorithms across these studies was influenced by^72^ (1) data availability and dimensionality: ANN and GPR were preferred for large, high-dimensional data sets.^73^ (2) Model interpretability requirements: fuzzy logic models (ANFIS) and ensemble trees (GBRT) were favored for processes where transparency and explainability were important.^17^ (3) Computational efficiency needs: LR and SVM were often selected for quick deployment in systems with simpler relationships.^17^ (4) Process complexity and nonlinearity: Deep learning and hybrid models were reserved for systems requiring advanced pattern recognition or multi-objective optimization.^72^

The performance of various ML models differs because they deal with aspects of nonlinearity, uncertainty, and complexity of data differently.^73^ ANNs are effective in modeling nonlinear systems, as is found in hydrogen production, but can sometimes lead to overfitting when not properly tuned. While GPR is probabilistic, which aids in noise handling for biological data, SVM is often outperformed by it. Hybrid models such as ANFIS-PSO and GBRT provide increased predictability, combining the strengths of learning and optimization but at a greater computational cost. Since gas quality estimation is an area surrounded by structured, low-noise data sets, simple models like Linear Regression tend to do well.

In all studies, artificial neural networks (ANN) typically used 2–4 hidden layers, each with 5–15 neurons, trained by gradient descent, Levenberg–Marquardt, Bayesian regularization, etc. Some studies included dropout layers or early stopping in their models to prevent overfitting.^17^ Support Vector Machines (SVM) were typically parametrized with a radial basis function (RBF) kernel, but the C and gamma hyperparameter settings had strong impacts on the performance of the SVM models. Some studies included hybrid models, such as ANFIS-PSO models that utilized fuzzy logic with particle swarm optimization to dynamically tune the membership functions, increasing accuracy and interpretability.^74^ Some studies used ensemble approaches, such as Gradient Boosted Regression Trees (GBRT), to reduce variance and improve the generalizability of their models when considering multiple inputs.^72^

In summary, the ANN remains the most versatile and widely used algorithm across all application areas due to its proven predictive accuracy and ease of adaptation. GPR and hybrid models provide enhanced performance, particularly when modeling uncertainty or when higher interpretability is required. For cost-sensitive or industrial applications, integrating ML with optimization algorithms such as GA or NSGA-II provides robust, actionable insights. The comparative analysis strongly supports the conclusion that ML techniques not only outperform traditional modeling approaches but also are essential for driving innovation in hydrogen production technologies. Table 4 presents the comparison of different machine learning models used along with their applications, the input and output variables with the best-performing model, and key observations.

**Table 4:** Structured Comparative Summary of Machine Learning Applications in Hydrogen Production

application area	ML techniques used	typical inputs	outputs predicted	best performing models	*R* ^2^	key observations
hydrogen yield prediction	ANN, GPR, SVM, DL, ANFIS, BANN, GBRT, PSO	pH, temperature, catalyst weight, feedstock composition, solar radiation, pressure, residence time	H_2_ yield, production rate, syngas composition	ANN, GPR, ANFIS-PSO, BANN	0.95–0.999	ANN most used; GPR outperformed SVM in biomass/waste; hybrid models (ANFIS-PSO) yielded better accuracy for nonlinear bioprocesses
cost and efficiency estimation	ANN, ANFIS, GA, NSGA-II, DL	temperature, flow rate, steam/carbon ratio, ambient conditions, plant size, reactor specs	H_2_ cost/kg, total annual cost (TAC), exergy efficiency, thermal/electrical efficiency	ANN-GA, ANFIS-NSGA-II, DL	≈0.999	ANN and ANFIS with optimization tools gave high accuracy; essential for techno-economic modeling in solar/geothermal/electrolysis systems
gas quality assessment	ANN, LR, SVM, DTR, KNN	time, gas composition (CH_4_, CO, CO_2_, O_2_), temperature, pressure, steam/carbon ratio	H_2_ concentration, H_2_ mole fraction, CO mole fraction	LR, ANN	0.98–0.999	LR effective for simple systems; ANN favored in industrial setups; ensemble methods used, but not always superior

## Current Challenges and Future Prospects

Hydrogen, being one of the cleanest energy carriers with a very high calorific value, should have replaced fossil fuels by now, but there are certain challenges that hinder the widespread adoption of hydrogen as the main fuel for global needs. These challenges include both costs and technical and policy aspects. The methods currently used for hydrogen production are mostly dependent on fossil fuels, which is why research and development for green hydrogen production is of the highest importance.^15^

A key restriction in the application of ML is the absence of standardized large open-access data sets for hydrogen production.^75^ Most of the studies made use of experimental data sets, and laboratory-scale processes (e.g., batch fermentation reactors, electrolyzers) were the sources of the experimental data sets. Another data source used was plant sensor data from industrial facilities.^76^ For instance, the GPR and ANN models were trained using data from pilot-scale biomass gasification to establish hydrogen production (e.g., the pressure, temperature, and ingoing gas composition). Public repositories that contain time-series data, thermodynamics data, and biochemical data would substantially speed up the development of ML in this field.

For hydrogen to be globally adopted as the primary fuel, development and investments are required for infrastructure, i.e., hydrogen storage, transportation, filling stations, etc.^6,77^ Also, it is very much dependent on government policies to promote hydrogen as a clean source of energy, having a negligible impact on the environment.^78^ The cost of hydrogen production is also very important in this regard as the global target is to reduce this cost to $1–2 per kg by 2030, which cannot be realized without advancements in knowledge, industrial collaborations among countries and the integration of modern techniques like machine learning to improve efficiency and make the processes faster and easier than before.^77^ Similarly, the role of policy frameworks and financial incentives is critical in scaling AI-integrated hydrogen production. Government-backed initiatives—such as tax credits, carbon pricing, hydrogen roadmaps, and innovation funding—can accelerate adoption by reducing investment risk. For example, national hydrogen strategies in the EU, Japan, and the U.S. increasingly emphasize digitalization and AI as enablers of clean energy systems.^78^

On the other hand, deploying AI/ML solutions in hydrogen production is often constrained by data quality and availability—industrial plants rarely maintain the large, standardized, labeled data sets needed to train robust models, and proprietary concerns can further limit data sharing.^75^ Moreover, significant computational demands require high-performance GPUs or cloud services that increase both capital expenditure and ongoing energy costs. Furthermore, successful implementation demands cross-disciplinary expertise: teams must combine deep process-engineering knowledge with data-science skills to curate data, select appropriate models, and validate their outputs against real-world performance.^73,76^ At the Industrial Scale, AI adoption encounters additional organizational and regulatory hurdles. Lack of digital infrastructure, real-time sensors, IoT connectivity, and automated control systems—necessary for seamless ML integration—and retrofitting legacy equipment can be prohibitively expensive. Cybersecurity and data-privacy concerns further complicate the use of cloud-based analytics or federated learning approaches.^78^ Finally, the absence of clear standards or regulations governing AI-assisted control in chemical plants, coupled with a skills gap in the current workforce, can slow pilot projects and delay the scaling of proven AI applications to full-scale industrial operations.

Based on the reviewed literature, future research at the intersection of hydrogen production and machine learning should consider the following important directions:

- Development of hybrid and interpretable models: ANNs and deep learning are largely utilized in studies to date. In future work, researchers should work toward an interpretable hybrid model (such as ANFIS, GPR + optimization) to provide accuracy and transparency when looking to deploy a model in an industrial context.^24^
- Integration with real time systems: From a research perspective, the recent advances in reinforcement learning and digital twin frameworks need to be matched with more real-time monitoring and adaptive control systems for hydrogen plants so that dynamic process and data management can take place.^24^
- Data standardization and open access: Developing high-quality standardized data sets across various hydrogen production concepts will be a vital step for modeling with ML that has reliability and accountability testing.^21^
- Scaling ML applications in green hydrogen: As it stands, the majority of ML applications still exist in a lab or pilot stage, so translating research models to full-scale electrolysis, biomass, and photobiological systems represents a major research gap.^9^
- Techno-economic and environmental optimization: There is a need to better integrate process input parameters, life cycle analysis (LCA), cost modeling, and environmental indicators into future models for the holistic optimization of hydrogen systems.^6^

## Conclusion

Human activity has long contributed to environmental degradation, but the increase in fossil fuel exploitation has peaked, reaching a critical level, necessitating a shift toward renewable and sustainable energy sources. This review underscores the potential of hydrogen as an exceptional energy carrier—boasting high calorific value and versatility for both transportation and industrial applications. Although current hydrogen production predominantly relies on fossil fuels through methods such as steam methane reforming, autothermal reforming, and partial oxidation, these approaches are insufficient to address the urgent climate change challenge. In contrast, green hydrogen production from renewable resources—particularly via water electrolysis and thermochemical water splitting—offers a promising, low-impact alternative. This review highlights three core insights: (1) hydrogen production methods vary significantly in efficiency and environmental impact, with biological and thermochemical routes requiring different optimization strategies; (2) ML, particularly ANN, GPR, and hybrid models, consistently enhance predictive modeling across production, cost, and quality dimensions; and (3) integrating ML into hydrogen systems not only improves performance but also accelerates the transition toward economically viable, sustainable hydrogen technologies. Furthermore, the machine learning (ML) has emerged as a pivotal strategy to optimize these renewable processes by enhancing the yield predictions and overall production efficiency. Moving forward, sustained research, development, and robust policy initiatives in hydrogen production, utilization, storage, and transportation are imperative for transitioning to a cleaner and more sustainable energy future.
